# Can gene editing reduce postharvest waste and loss of fruit, vegetables, and ornamentals?

**DOI:** 10.1038/s41438-020-00428-4

**Published:** 2021-01-01

**Authors:** Emma N. Shipman, Jingwei Yu, Jiaqi Zhou, Karin Albornoz, Diane M. Beckles

**Affiliations:** 1 Department of Plant Sciences, University of California, Davis, CA 95616 USA; 2 Plant Biology Graduate Group, University of California, Davis, CA 95616 USA; 3 Graduate Group of Horticulture & Agronomy, University of California, Davis, CA 95616 USA; 4 Departamento de Produccion Vegetal, Universidad de Concepcion, Region del BioBio, Concepcion, Chile

**Keywords:** Molecular engineering in plants, Molecular engineering in plants

## Abstract

Postharvest waste and loss of horticultural crops exacerbates the agricultural problems facing humankind and will continue to do so in the next decade. Fruits and vegetables provide us with a vast spectrum of healthful nutrients, and along with ornamentals, enrich our lives with a wide array of pleasant sensory experiences. These commodities are, however, highly perishable. Approximately 33% of the produce that is harvested is never consumed since these products naturally have a short shelf-life, which leads to postharvest loss and waste. This loss, however, could be reduced by breeding new crops that retain desirable traits and accrue less damage over the course of long supply chains. New gene-editing tools promise the rapid and inexpensive production of new varieties of crops with enhanced traits more easily than was previously possible. Our aim in this review is to critically evaluate gene editing as a tool to modify the biological pathways that determine fruit, vegetable, and ornamental quality, especially after storage. We provide brief and accessible overviews of both the CRISPR–Cas9 method and the produce supply chain. Next, we survey the literature of the last 30 years, to catalog genes that control or regulate quality or senescence traits that are “ripe” for gene editing. Finally, we discuss barriers to implementing gene editing for postharvest, from the limitations of experimental methods to international policy. We conclude that in spite of the hurdles that remain, gene editing of produce and ornamentals will likely have a measurable impact on reducing postharvest loss and waste in the next 5–10 years.

## Introduction

Plant gene editing may be the greatest innovation in plant breeding since the Green Revolution. It has already been used to make discoveries in plant biology and has a profound potential to create new crops with desirable characteristics^1^. There are already exciting developments, which show that gene editing may be able to live up to expectations and can be used to produce novel plant phenotypes that would improve agricultural production.

Most authorities estimate that food production will have to double in the next 50 years to keep pace with population growth^2^. The focus on global food security, however, is usually on starch-rich cereals and ignores or underestimates the vital importance of horticultural crops. These perishable commodities are often nutrient-dense with bioactive phytochemicals, the consumption of which is needed for a healthy and thriving population^3–6^. However, an uncomfortable fact is that in addition to losses that may result from disease, drought, extremes of temperature, and other environmental stresses experienced in the field, an additional 25–40%—an average of 33%—of all fruit and vegetables produced globally are never eaten after harvest^7^. This estimate still does not illustrate the extreme losses that can occur in some developing countries, which may be as high as 75%^8,9^. Current worldwide horticultural crop production is insufficient to meet human nutritional requirements, making postharvest loss and waste all the more unsustainable^10^. Only recently has the need to reduce the loss of horticultural crops after harvest been given the attention it deserves^7–9,11–14^.

Although the causes of postharvest loss and waste are complicated, we suggest that technology-assisted breeding for new and improved fruit, vegetables, and ornamentals, compatible with supply chain constraints but delivered at peak quality to the consumer, could be an important part of the solution over the long-term. In this review, we examine the potential for gene editing to make a measurable and robust impact on postharvest waste and loss. Rather than a technical or critical assessment of methodologies or research areas, we focus on connecting the bio-physiology of postharvest produce, the needs of the produce industry, and the wealth of existing molecular research, to suggest a holistic yet straightforward approach to crop improvement. The main focus of the review is the discussion of genes that could influence the quality and shelf-life of produce. First, we examine the steps that are taken to extend shelf-life in the produce supply chain, and the impact of supply chain management on consumer-desired quality traits. Then we briefly review the CRISPR–Cas9 method to emphasize the flexibility, ease, and power with which traits can be modified. Finally, we take a critical look at remaining barriers which must be overcome to make gene editing for postharvest traits technically and economically viable. This review serves both as an introduction to postharvest and gene editing and as a resource for researchers attempting to utilize the latter for the former.

## Overview of postharvest loss and waste (PLW)

Postharvest waste and postharvest loss are sometimes used interchangeably, but this is incorrect. Postharvest loss is *unintentional*. It describes the incidental losses that result from events occurring from farm-to-table, such as physical damage, internal bruising, premature spoiling, and insect damage, among others. Produce loss is also described as *quantitative* because it is measurable. This does not imply that data is easily available, only that it can be assessed^8,12^.

Postharvest waste, in contrast, is *intentional*. It describes when produce is discarded because it does not meet buyer expectations, even though it is edible^8,12^. Produce may be rejected by growers, distributors, processing companies, retailers, and consumers for failing to meet desired or established preferences. Produce waste is described as *qualitative* because it is difficult to measure and assess^8^. Still, in the US, it is estimated that 7% of postharvest *losses* of fruit and vegetables occur on the farm, while more than twice that, i.e., 17% and 18% are *wasted* in consumer-facing businesses and in homes, respectively^14^.

Produce postharvest loss and waste (PLW) threatens environmental sustainability, and is especially catastrophic when viewed in the light of the twin challenges of global climate change and increasing population growth. PLW means inefficient use of financial investments in horticulture and more critically, non-renewable natural resources. Technological measures to curb PLW, such as maintaining a cold-chain and use of plastic packaging, additionally have energy and carbon costs. Improving the shelf-life and quality attributes of postharvest crops by genetic modification or smart breeding could be among many solutions to lessen the severity of these problems.

## The challenge of the postharvest supply web

**Fig. 1:**
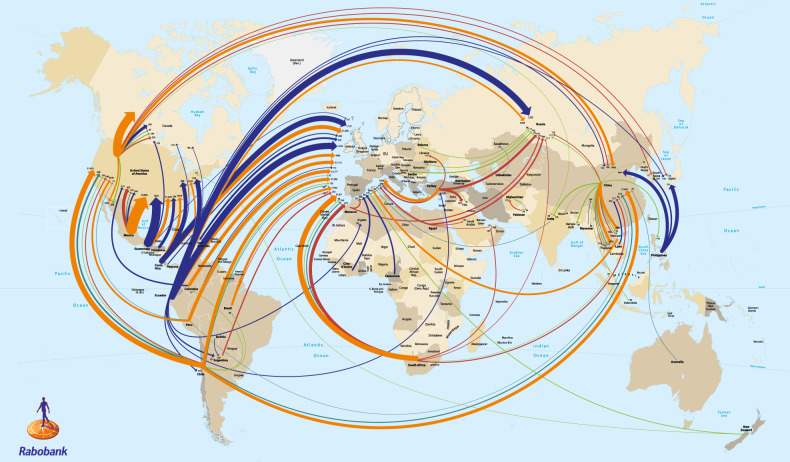
Map of the global trade of fruit in 2016. An estimated 80% of all fruits grown globally are sold as whole fresh fruit. Key to colored lines: orange—trade movement and monetary value of total fresh fruit, excluding nuts and frozen fruit. Other lines illustrate commodity volume. Blue—bananas and plantains; green—apples; aquamarine—grapes; red—citrus. The minimum requirement for trade values to be shown is USD 500 million, while for commodity volume, it was bananas, plantain, and citrus—100,000 tons and for apples and grapes—50,000 tons. Line thickness proportional to the magnitude of the trade. Source: van Rijswick^273^ RaboResearch, The Netherlands. Reproduced with the kind permission of RaboResearch

Produce must be kept alive from farm to table; however, the biological nature of horticultural produce is often incongruent with modern commercial supply chain operations^15^. Produce and ornamentals are high in water content, and often metabolically active, which makes them highly perishable^15–17^. This becomes a challenge given the number of food miles fruit, vegetables, and ornamentals can travel in the global supply chain (Fig. 1).

Modern postharvest supply chains may be separated spatially by thousands of miles, and temporally, by several months. Produce trucked and shipped from the field is often treated: cooled, washed, sorted, dipped, sprayed, or held at desirable temperatures and modified atmospheres to preserve “health”. The majority of produce from mid- to large-scale operations may move through a byzantine system of processors, distributors, and trucking and shipping entities. Maintaining an unbroken cold-chain, adequate packing, and shipping are essential to preserving quality and shelf-life. (Zoom in on map to read text).

**Fig. 2:**
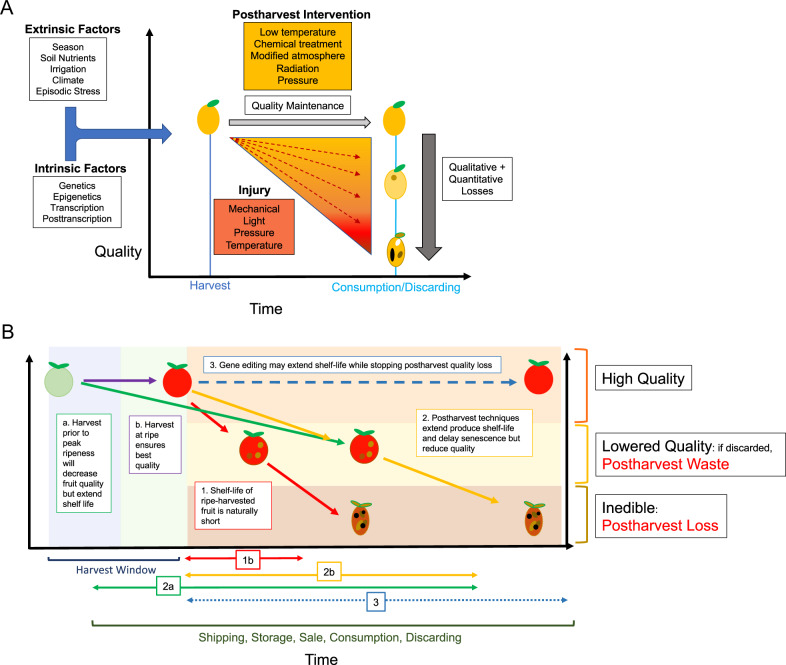
Determinants of produce quality. **a** Extrinsic environmental factors such as season, irrigation, soil nutrition and minerals, climate, stress, pathogens and pests, and agronomic practices as well as physiological genetic factors together determine fruit quality at harvest. Postharvest intervention, including refrigeration, chemical treatment, radiation, and modified atmospheres and pressure aims to maintain that quality through shipping and storage. Minor injury, ranging from mechanical or pathogenic damage to temperature, light, or pressure-induced damage, lowers the quality of fruit. More extensive injury renders produce inedible and contributes to the quantitative loss. **b** Potential postharvest outcomes for produce. Harvesting fruit prior to full ripeness will increase its shelf-life [a], but compromises quality during and after ripening [2a]. Fruit harvested at ripe [b] has a limited shelf-life before it declines in quality or rots [1b]. Postharvest intervention delays senescence and typically also results in some compromise of quality [2b]. The goal of gene editing is to extend shelf-life without loss of quality [3] and therefore reduce postharvest loss and waste

Produce, even after harvest, respires (taking up oxygen and producing carbon dioxide), transpires water, and, for the “climacteric fruits”, can emit high levels of ethylene, which can be accelerated at high temperatures. Optimizing storage and handling conditions requires managing these biological processes (Fig. 2), which may differ for each produce-type or variety, and from how the preharvest environment influences biology at harvest and thereafter^15^. Temperature, humidity, ethylene levels, and the storage oxygen-to-carbon dioxide ratio must be controlled to slow down maturation and senescence in order to maintain produce shelf-life and quality^15,18,19^. Low temperatures are used to reduce respiration, thereby extending shelf-life^18^, but also have the added benefit of suppressing water loss, shrinkage, and fungal growth, which can occur due to physical injury and physiological disorders^18,20^. Modifying the atmosphere to change the carbon dioxide-to-oxygen ratio and relative humidity using modified atmosphere packaging or large-scale storage of produce in controlled atmosphere rooms can extend the postharvest life of commodities (Fig. 2).

Ethylene biosynthesis and emission underpin postharvest quality and shelf-life in climacteric fruit^21–24^ and vegetables^24–27^. Ethylene accelerates ripening, but also senescence; therefore, ethylene must be managed to optimize shelf-life. This is underscored by the number of ethylene inhibitors, absorbers, and blockers^18^ on the market (Fig. 4).

The biological reality of ripening is that its natural end is senescence. The goal of postharvest management is therefore to control this progression to senescence, i.e., to pause the ripening process for shipping and storage, and then to restart it with a minimal loss of quality. However, the processes that control the ripening-to-senescence transition dictate quality, creating a dilemma, whereby altering ripening biology via refrigeration, chemicals, or other means to lengthen shelf-life, often unavoidably disrupts ripening outcomes and reduces quality^28^. This leads to consumer rejection and postharvest waste. The alternative—to maximize consumer preference by harvesting produce close to peak maturity stage, and with no chemical or physical treatment, will invariably increase postharvest losses due to the shortened shelf-life, and increased susceptibility to bruising and pathogenic infection (Fig. 2).

## Potential for improving postharvest quality of horticultural crops by gene editing

There is great excitement at the innovation gene editing and the associated technologies potentially bring for improving crop quality, especially for species and traits that have been relatively understudied, such as postharvest traits of horticultural crops. Manipulation of plant genomes in a precise manner has been achieved at a spellbinding pace since the era of genome editing^29–31^. The current gene-editing tool of choice is CRISPR–Cas9. The researcher is able to generate mutations in narrowly defined regions of the genome, and it has been successfully applied to induce valuable traits in many crop species^32,33^. Further, CRISPR can bypass other burdens like sterility, self-incompatibility, high heterozygosity, low frequency of recovering desired alleles and traits and long life cycles, which extend or halt entirely conventional breeding efforts^34–36^.

CRISPR is a prokaryotic system that protects organisms from viral infection^37^. This naturally occurring mechanism in bacteria has been co-opted by scientists to remove unwanted nucleotides or to insert new or altered ones to promote traits seen as desirable in an organism of interest. For CRISPR editing, a synthetic guide RNA (gRNA) is designed to an identified protospacer adjacent motif (PAM) in the sequence of interest, and this, along with the Cas protein sequence, is inserted into a cell where they are processed using the cell’s gene expression apparatus. The Cas protein synthesized in the plant produces a double-stranded break (DSB) at the bases identified by the gRNA. Repair of the DSB in DNA is usually not faithful to the original sequence, and thus, non-synonymous mutations may be introduced into the genome. The precise changes in nucleotide sequences are difficult to predict, but indels (insertion–deletions) of varying sizes and single-nucleotide polymorphisms are most common, providing diverse genetic variants^38^. DSB repairs occur naturally in almost all plant tissues, so this is not an inherently foreign process^39,40^.

Although genomic mutations generated by CRISPR-mediated random repair mechanisms are easily achieved, the ability to specifically express the Cas protein in a controlled spatial-temporal manner, and in conjunction with other enzymes, is often desirable for basic and applied plant research. Precise site-directed editing can be used for single-base substitution of a gene(s) of interest^41^, which has been achieved in cereals^42,43^, as well as horticultural crops such as tomato and potato^42,44,45^. In addition, tissue-specific knockouts using a CRISPR technique, called CRISPR-TSKO, can generate somatic mutations in cells, tissues, and organs by using specific promoters^46^. Similarly, another gene-editing system uses an inducible chimeric transcription factor (XVE), to control the expression of Cas protein in planta^47,48^.

Apart from knock-out/in of gene coding regions, transcriptional modulation of gene expression can be achieved by CRISPR targeting of gene regulatory elements^49^. New alleles generated by CRISPR/Cas in promoters and enhancers where transcription factors (TFs) bind to direct gene expression, can lead to fine-tuned expression^1,50,51^. Similarly, variants in upstream open reading frame (uORFs) sequences could enhance post-transcriptional modulation of gene expression, influencing phenotype^1^.

The expression of a gene may also be varied by changing its DNA methylation status. In tomato, orange, and bell pepper^52–54^, DNA methylation regulates ripening by controlling ripening-related TFs or genes. Binding a methylation modifying protein to a CRISPR complex with a deactivated Cas9^55^ may be a feasible approach to edit regions targeted for de/methylation in ripening-related genes, thus controlling shelf-life.

CRISPR-Cas also enables modulation of traits in species that are difficult to obtain through traditional breeding. Approximately 70% of angiosperms are polyploid, which increases the effort needed for introducing new alleles by crossing and selection^56^. Transmission of Cas activity in the progeny of CRISPR-expressing lines holds promise for transgenerational gene-editing in polyploid plants. This method was shown to introduce newly mutated alleles, not only in F_1_ but also in F_2_ and F_3_ plants^56,57^. De novo domestication, a new idea in crop improvement, has been demonstrated in multiple species of the wild *Solanum* genus by CRISPR targeting^58^. Novel alleles of selected “domestication genes” are generated in wild species, landraces, or non-commercial genotypes to speed-up their transformation to elite varieties suitable for cultivation and postharvest practices of modern agriculture^1,50,51^.

In conclusion, various CRISPR techniques and approaches can be used to introduce nuanced changes in the expression of single or multiple genes, however, it also has real value as a tool to dissect the network of biological pathways responsible for ripening, senescence, and quality. It is expected to help identify hitherto unknown genes, that when altered, can promote favorable postharvest phenotypes. These desirable phenotypes are discussed in “Produce postharvest attributes that would minimize PLW” section.

## Produce postharvest attributes that would minimize PLW

**Fig. 3:**
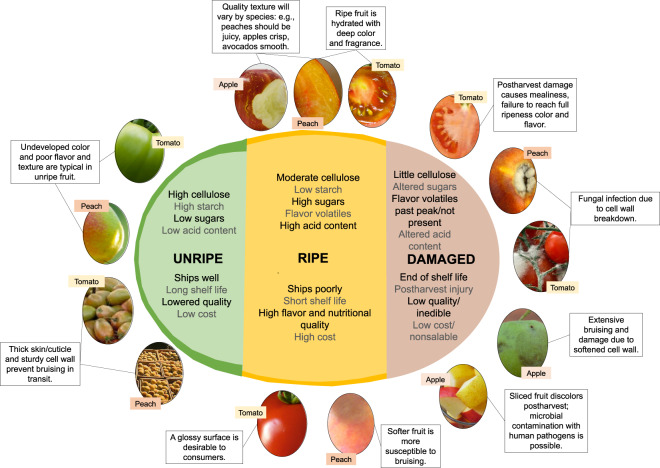
Linking quality to physio-biological characteristics. The physiological factors that confer produce quality (flavor, texture, shelf-life, aroma) are determined by the amount and interactions of metabolites, both primary and specialized, present in the various tissues. Texture and shelf-life are tightly connected to the cell wall and cuticle integrity, while flavor and aroma are linked to levels of sugars, acids, and other metabolic products. Biological changes in these factors due to the natural ripening-senescence transition or, due to postharvest handling, determine consumer acceptance

Recent consumer trends indicate a growing interest in consuming fruit and vegetables for their nutritional value. This is especially notable for middle-class consumers of emerging economies. Gene editing to reduce PLW may improve the overall efficiency of fruit and vegetable production so that costs may be lowered^59^, thereby bringing fresh produce within the means of more populations^60–64^ and strengthening the industry as well as worldwide health. There is also a demand for the produce of exceptional quality among discerning consumers^65,66^, a rising interest in organic and locally sourced produce, and in semi-prepared or “fresh-cut” vegetables and fruit^16,17,67,68^ that are reasonably priced. Key attributes are outlined below (Fig. 3):

### Longer shelf-life with maximal quality retention

Many of the approaches for extending the life of produce often lead to poor taste and flavor, and this link must be broken to increase consumer satisfaction and repeat purchases^68,69^.

### Convenience

The fresh-cut industry has grown over the last 20 years, driven by a demand for convenience. The ability to eat fruit and vegetables directly from the packet has been a boon to the produce industry^70^. Quality attributes needed to provide safe, long-lasting, visually and texturally appealing fresh-cut products can be challenging to maintain since cut produce often respires faster and is prone to browning and premature senescence^70^. Microbial contamination, especially of fresh-cut leafy greens and fruit by *E. coli*, *Salmonella*, and *Listeria* is also problematic^71^.

### Better quality

Consumers have shown that fruit and vegetables with desirable appearance, texture, taste, and flavors will have higher salability^16,17,65,67^. The criteria for a favorable appearance include produce of the right color and color uniformity, correct shape and dimensions, and often a glossy surface area free from defects^15,67,70^ (Fig. 3). Identifying and manipulating the genes determining these pathways could improve quality. Consumers also have specific notions of what “unacceptable” produce is, and this has consequences for the generation of postharvest waste. This may vary culturally and according to socio-economic status, but general trends are identifiable. Produce with characteristics reminiscent of rotten, infested, or unripe material will be rejected. This is widely accepted as an evolutionary strategy to avoid poisoning or illness from contaminated food^72^, as well as a learned response based on a previous negative experience. Therefore, lesions or aromas due to age or bruising are associated with “bad” fruit and vegetables and will be rejected not only as “low quality” but as potentially dangerous, despite the produce being largely intact and actually safe. While quality of flavor is widely believed to be a strong predictor of repeat purchase^73^, visible appearance has a strong role in initial selection or rejection at the point of purchase, and later discarding in the home^74,75^. These negative traits all interact with the consumer priorities mentioned above and contribute to postharvest waste.

## Biological processes “ripe” for editing—shelf-life

Although our knowledge of basic fruit and produce biology is incomplete, there has been extensive work that points to the action of individual genes which, when altered in expression, may deliver useful phenotypes. Manipulating these biological processes by gene editing is a promising new avenue for reducing PLW. Many traits, however, are determined by networks of genes, and although distinct, some networks overlap so that changes in one may have unintended consequences in another. A major challenge is to understand the complicated regulation of these pathways in order to fine-tune them in a beneficial way. Gene editing has the potential to clarify the role of individual constituents in conjunction with the production of novel varieties.

### Ethylene production

**Fig. 4:**
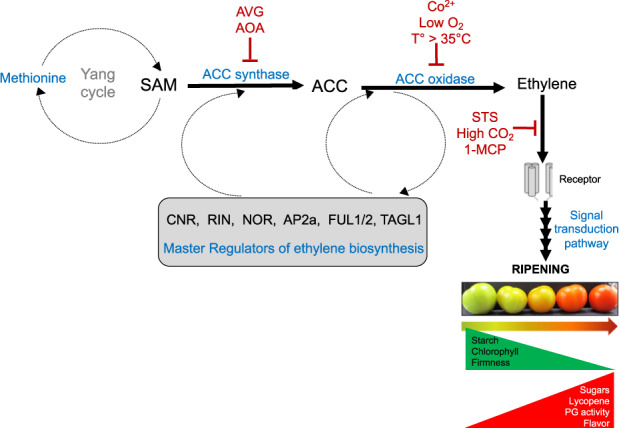
Genetic determinants of ethylene production and their control for extended postharvest shelf-life. Ethylene biosynthesis occurs in two enzymatic steps, catalyzed by ACC (1-aminocyclopropane-1-carboxylic acid) synthase (ACS) and ACC oxidase (ACO)^274^. In climacteric fruit, such as tomato, banana, mango, and apple, there is a rapid rate of increase in ethylene at the onset of ripening and continued production leads to ripening and senescence. Enzymatic inhibitors AVG (aminoethoxyvinylglycine) or AOA (aminooxyacetic acid) inhibit ACS^275^. Cobalt ions (Co^2+^), high temperatures (*T*°), and low oxygen concentration inhibit ACO or low oxygen concentration inhibits ACO^276^. Silver ion, silver thiosulfate (STS), potentially, carbon dioxide, and 1-methylcyclopropene (1-MCP) inhibit ethylene binding to the receptor for activation of ethylene signaling pathway^277,278^. For example, 1-MCP, a synthetic growth regulator structurally related to ethylene, is commercially used in fruit crops such as apple, kiwifruit, pear, avocado, melons and others, and it has also shown biological benefits in a range of other species^16,279^. SAM *S*-adenosyl methionine

As mentioned in “The challenge of the postharvest supply web” section, ethylene is a master regulator of ripening; in climacteric fruit, ethylene production must be managed to optimize shelf-life (Fig. 4), but genetic solutions may be more effective. In climacteric fruit, ethylene synthesis, regulation, and perception lead to the transcription of ripening-regulated genes that determine quality attributes desired by consumers. When *ACO* and *ACS* (Fig. 4) expression is genetically suppressed or silenced in a range of species, e.g., petunia, tomato, melon, papaya, and kiwifruit, ethylene production is decreased and shelf-life is extended due to slowed ripening processes^76–83^.

In tomato, the regulation of ethylene biosynthesis is mediated by a complex network centered around the master regulatory proteins: CNR, RIN, and NOR, which are required for normal ripening^84^. The recent use of CRISPR to induce targeted deletions or substitutions in *CNR*^53^ and *NOR*^53,85^, and in other transcription factors (Fig. 4), *AP2a*, *FUL1*, and *FUL2*^86,87^ revealed multiple and redundant levels of regulation in the ripening pathway. Using CRISPR to create fruit varying sequentially in one or more of these transcription factors may improve our understanding of the molecular regulation of ethylene response in horticultural crops. This knowledge would allow us to control ethylene production so that ripening proceeds at the rate and with the timing that is optimal for supply chain dynamics while maintaining quality. This would directly mitigate PLW.

### Flower vase-life

Global demand for fresh-cut ornamentals has increased in the past years, with an estimated value of $16B in 2015^88^. The top producers, the Netherlands, Ecuador, Columbia, and Kenya, export floral products long distances, primarily to Europe, North America, and East Asia^88,89^. However, ornamental crops are highly perishable and up to 50% of the farm value may be lost along the cold-chain^90^, and each extra day in transit leads to a 15% loss of value^91^. Further, after consumer purchase, ornamental shelf-life, i.e., vase-life, is typically only 10–12 days^91^, so rapid transport along a cold-chain is essential^91,92^.

Ethylene has a critical role in accelerating flower senescence in some species, and targeting components of the ethylene signal transduction pathway has been successful in extending vase-life in carnation^93–95^ and petunia^54,96–98^ (Table 1). Gene editing was also used to mutate *ACO1* in petunia thereby increasing flower longevity^99^. In species that are not ethylene-responsive, vase-life could also be extended by inhibiting general senescence proteins^100^.

**Table 1:** Targets for improved postharvest quality and enhanced shelf-life

Species	Trait	Gene	Change*	Method	Phenotype	References
Tomato (*Solanum lycopersicum*)	Shelf-life	*SlFSR*	↓	RNAi	↓ Expression of cell wall modification enzymes in fruit	Zhang et al.^49^
Tomato (*Solanum lycopersicum*)	Shelf-life	*SlACO1*	↓	RNAi	↓ Ethylene, ↓firmness loss associated with ↓ PME and PG activities	Behboodian et al.^261^
Tomato (*Solanum lycopersicum*)	Shelf-life	*PG*	↓	Antisense RNA	↑ Fruit firmness, ↓ postharvest fungal infection	Kramer et al.^262^
Strawberry (*Fragaria* × *ananassa*)	Shelf-life	*FaPG1*	↓	RNAi	↑ Soluble solids, firmness and ↓ softening	Quesada et al.^263^
Tomato (*Solanum lycopersicum*)	Shelf-life	*Del, Ros*	↑	Ectopic Expression	Double shelf-life and ↓ susceptibility to *Botrytis*	Zhang et al.^143^
Tomato (*Solanum lycopersicu*m)	Shelf-life	*Aft, atv*	–	Breeding	Extended shelf-life ↓ susceptibility to *Botrytis*	Bassolino et al.^145^
Tomato (*Solanum lycopersicum*)	Shelf-life	*SlALC*	↓	CRISPR/Cas9	Extended shelf life	Yu et al.^85^
Tomato (*Solanum lycopersicum*)	Ripening	*SlPL*	↓	CRISPR/Cas9	↑ Firmness, no change in color	Wang et al.^260^
Banana (*Musa acuminata*)	Ripening	*MaMADS1 MaMADS2*	↓	RNAi	Delayed ripening	Elitzur et al.^264^
Eggplant (*Solanum aethiopicum*)	Color and nutritional content	*SmMYB1*	↑	Overexpression	↑ Anthocyanin: flesh and peel	Zhang et al.^190^
Tomato (*Solanum lycopersicum*)	Softening	*SlPG*	↓	Antisense RNA	↓ Pectin depolymerization	Smith et al.^127^
Tomato (*Solanum lycopersicum*)	Softening	*SlPL*	↓	RNAi	↑ Firmness, cellulose, hemicellulose, ↓ soluble pectins	Yang et al.^265^
Tomato (*Solanum lycopersicum*)	Softening	*PME*	↓	Antisense RNA	↓ Soluble pectins and polyuronides, ↑ soluble solids	Tieman et al.^266^
Tomato (*Solanum lycopersicum*)	Softening	*DkGal1*	↑	Heterologous expression	↓ Cell-to-cell adhesion, ↑ ripening rate, ↑ ethylene production	Ban et al.^267^
Tomato (*Solanum lycopersicum*)	Softening	*TBG4*	↓	Antisense RNA	↑ Firmness	Smith et al.^123^
Tomato (*Solanum lycopersicum*)	Softening	*PE1, PE2 (PME)*	↓	Antisense RNA	↓ De-esterification of pectins	Wen et al.^122^
Strawberry (*Fragaria* × *ananassa*)	Softening	*FaPG1*	↓	Antisense RNA	↑ Firmness, reduced soluble pectin, ↑ cell integrity	Posé et al.^115^
Strawberry (*Fragaria* × *ananassa*)	Softening	*FaPL*	↓	Antisense RNA	↑ Firmness, ↓ soluble pectins	Jiménez-Bermudez et al.^117^
Strawberry (*Fragaria* × *ananassa*)	Softening	*FaPL*	↓	Antisense RNA	↓ Soluble pectins, ↑ cell-to-cell adhesion	Santiago-Doménech et al.^118^
Strawberry (*Fragaria* × *ananassa*)	Softening	*FaβGal4*	↓	Antisense RNA	↓ Softening, ↓ soluble pectins	Paniagua et al.^268^
Tomato (*Solanum lycopersicum*)	Ripening	*RIN*	↓	CRISPR/Cas9	Slower ripening	Ito et al.^87^
Tomato (*Solanum lycopersicum*)	Ripening	*LeCTR1, LeEILs LeEIN2*	↓	VIGS	Ripening suppression	Fu et al.^269^
Petunia (*Petunia* × *hybrida* cv. “Mitchell diploid”)	Senescence/vase-life	*Atetr1-1*	↑	Ectopic expression	Doubled vase-life	Wang et al.^98^
Petunia (*Petunia* × *hybrida* cv. Hort. Vilm.-Andr.)	Senescence/vase-life	*BoACO1, BoACS1*	↓	Antisense	Delayed flower senescence, extended vase-life	Huang et al.^270^
Petunia (*Petunia* × *hybrida* cv. “Primetime Blue” and cv. “Mitchell Diploid”)	Senescence/vase-life	*PhHD-Zip*	↓	VIGS	↑ Vase-life by 20%	Chang et al.^96^
Carnation (*Dianthus caryophyllus* L. cv. “Scania” and “White Sim”)	Senescence/vase-life	*ACO*	↓	Antisense	↑ Vase-life by 50%	Savin et al.^93^
Petunia (*Petunia hybrida* cv. Mirage Rose	Senescence/vase-life	*PhACO1*	↓	CRISPR	↑ Vase-life by 50%	Xu et al.^99^
Potato (*Solanum tuberosum*)	Shelf-life/quality	*StVInv*	↓	RNAi	↓ Acrylamide in fried chips	Bhaskar et al.^184^
Potato (*Solanum tuberosum*)	Shelf-life/quality	*StR1 (GWD), StPhL, StPPO*	↓	Antisense	↓ Acrylamide in fried chips, improved fry aroma	Rommens et al.^271^
Potato (*Solanum tuberosum*)	Shelf-life/quality	*StAs1, StAs2*	↓	Antisense	↓ Acrylamide in fried chips	Rommens et al.^272^
Potato (*Solanum tuberosum*)	“High fiber”	*StSBEI, II*	↓	CRISPR	↑ Resistant starch	Tuncel et al.^259^
Tomato (*Solanum lycopersicum*)	Sweetness	*SlbZIP1*	↑	Overexpression Fruit-promoter	50% ↑ in fruit sugar, no change in yield	Sagor et al.^155^
Tomato (*Solanum lycopersicum*)	Sweetness	*SlFgr*	↑	Wild allele & Overexpression	↑ Ratio of fructose to glucose, ↑ sweetness	Shammai et al.^156^
Tomato (*Solanum lycopersicum*)	Sweetness	*SlARF10*	↑	Overexpression	↑ Sucrose, fructose glucose not assayed	Yuan et al.^157^
Tomato (*Solanum lycopersicum*)	Sweetness	*AGPaseL*	↑	Allele from *S. habrochaites*	↑ Total sugars ↑yield	Petreikov et al.^151^
Tomato (*Solanum lycopersicum*)	Varied	*fumarase*	↓	Antisense RNA	↑ Sugars, ↓ infection by *Botrytis*, ↓ postharvest water loss ↓ yield	Centeno et al.^152^
Tomato (*Solanum lycopersicum*)	Sweetness	*Lin5 allele invertase*	↑	Natural allele	↑Sugar	Fridman et al.^150^
Tomato (*Solanum lycopersicum*)	Sweetness	*SlGLK2*	↑	Natural allele Transgenic line	↑ Sugars, ↑ lycopene yield same	Powell et al.^153^
Cucumber (*Cucumis sativus*)	Sweetness	*CmTST2*	↑	Overexpression	↑ Fructose, glucose, ↑ sucrose, yield unknown	Cheng et al.^158^
Strawberrry (*Fragaria × ananassa* Duch)	Sweetness	*CmTST2*	↑	Overexpression	↑ Fructose, glucose, ↑ sucrose, yield unknown	Cheng et al.^158^
Potato (*Solanum tuberosum*)	Appearance	*StPPO2*	↓	CRISPR	↓ Browning	Gonzalez et al.^139^
Apple (*Malus domestica*)	Appearance	*MdPPO*	↓	RNAi	↓ Browning	Okanagan Specialty Fruits Armstrong & Lane^199^
Potato (*Solanum tuberosum*)	Appearance health, texture	*StPPO* *StvINV* *StAsp* *StPHO* *StGWD*	↓	RNAi	↓ Browning ↓ Acrylamide ↓ CIS Better texture	Simplot Company, Richael et al.^185^

*Change in gene expression

### Fruit cuticle

The triterpenoids and waxes coating the harvested parts of horticultural crops may have a bigger influence on quality and shelf-life than previously recognized^101,102^. The plant cuticle is the first layer of defense against water loss and pathogen infestation^103^. The cuticle is also responsible for multiple traits involved in fruit quality and shelf-life, such as surface brightness^104^, the characteristic “bloom” of grapes^105^, blueberries^106^ and plums^107^, and potentially modulating texture changes^101^. Fruit cuticle composition actively changes depending on the environment and organ developmental stage, which affects its protective function during fleshy fruit ripening^108^.

The interaction between the biomechanical properties of the fruit cuticle and cell wall polysaccharides affects the development of surface cracks in cherries^109^, apples^110^, and tomato^111^. These aesthetically undesirable traits for consumers can also reduce produce shelf-life. Identifying genes key to cuticle compound biosynthesis could improve fruit response to environmental stresses during postharvest storage and reduce pathogen susceptibility.

### Fruit softening

The breakdown of the cell wall (CW) during fruit ripening is a crucial process in the development of fruit sensorial quality. Softening the fruit is essential for increasing its appeal to animals and humans for consumption, and thus seed dispersal^112^. Ripening and senescence, together with fungal attack, accelerate the rate of CW degradation, leading to rotting^113^. Rotting and ripening are discussed separately, even though they overlap biologically in relation to CW softening and fruit shelf-life. The modern, worldwide food supply chain often necessitates that the breakdown of the cell walls, either by ripening, senescent processes or by fungal rot, be halted or slowed.

CW softening processes are catalyzed by multiple enzymes that respond to developmental and environmental cues and occur over a variety of timelines, depending on the organism and tissue in question. CW degradation is orchestrated by polygalacturonase (PG), pectin methylesterase (PME), pectate lyase (PL), and β-galactosidase (β-Gal)^24,114^. PG, PME, PL, and β-Gal vary in their biotechnological potential to control firmness/fruit softening (Table 1). PG expression negatively influences firmness and shelf-life in strawberry,^115^ but only shelf-life in tomato^116^. In contrast, suppression of PLs reliably increases firmness and shelf-life in the species studied^117–119^. Suppression of PMEs^81,120–122^ and β-Gals^24^ promote fruit softening in many fruits. However, antisense downregulation of β-Gals in tomato caused cracking and other negative phenotypes^123–125^.

Managing the timing of these CW enzyme activities could support efforts to maintain the physical integrity of fruit and vegetables from farm-to-plate. Because of the cumulative and interactive effects of these enzymes^126,127^, it may be necessary to intelligently target single or multiple enzymes and their inhibitor proteins^128^ simultaneously, to create an optimal balance of CW degradation activities. Such efforts may make it possible to surmount, bypass, or control these complex interactions, and produce fruits that retain desirable textures but that show less softening in handling, shipping, and storage.

### Fungal rots

Harvested produce is susceptible to pathogenic attack. Invading fungi or bacteria will macerate the fruit components, creating rot: fruits covered in bacteria or spores, and their metabolic by-products. The result: commodities that are unsightly and also inedible due to a combination of the sour, bitter, putrid, or toxic compounds produced^129^. This may be advantageous in the spreading of seeds by attracting distributing animals or physically destroying CWs^130–132^ but is inconvenient for postharvest storage of commodities.

Fungal infections typically occur across the physical surface: the cuticle and cell wall. Therefore, all considerations in “Fruit cuticle” and “Fruit softening” sections impact susceptibility to pathogenic attack. One approach for reducing pathogen susceptibility could be to directly target plant CW and ripening-related processes^113,133^, which would also increase shelf-life by extending the integrity of the CW. Recent work in tomato shows targeting *PL* with gene editing may protect ripe fruit^134^.

Gene editing of endogenous plant enzymes that target fungal CW components and linkages could enhance resistance to fungal infection^135–137^. Further, the accumulation of specialized metabolites conferred pathogen resistance in several citrus species^138,139^ and could be a target for editing in others^140,141^.

## Composition

### Anthocyanins

Many of the red, blue, and purple colors seen in fruit, flowers, and tubers are due to anthocyanins. This class of compounds is not only aesthetically pleasing but has healthful antioxidant properties^142^. In addition, high-anthocyanin accumulation has been linked to increased shelf-life and reduced *Botrytis* infection in tomato. This was shown via ectopic expression of anthocyanin biosynthesis genes from snapdragon in tomato under a fruit specific promoter^143,144^, as well as by introgression of naturally occurring *Abg*, *Aft*, and *atv* high-anthocyanin alleles into tomato^145,146^. Increasing anthocyanin production in tomato and other fruit, could simultaneously enhance shelf-life for reduced postharvest loss, but also reduce postharvest waste by increasing the attractiveness of the fruit due to healthful properties and novel color.

### Carbohydrates

The primary carbohydrates studied are starch and sugars, and their interconversion, content, and relative amounts may influence postharvest quality. Starch breakdown to sugars is undesirable in potato (see “Cold-induced sweetening” section), but during maturation, it is valuable in several species, e.g., apple, banana, and kiwifruit. In others, e.g., sweet peas and sweet corn, the conversion of sugars to starch reduces sweetness and hence quality.

#### Sugars

Sweetness is an important attribute in fruits. Sweetness is determined by the concentration and relative ratio of the predominant sugars in fruit tissues^67,69^, although amino acids and other compounds may have an effect^147^. The biochemical pathways that lead to sugar accumulation have been studied, however, transgenic manipulation of these genes often has negative effects on yield^148^, indicating that a more fundamental and holistic knowledge of sugar metabolism, especially its regulation, is needed^149^. However, high sugar Quantitative Trait Loci (QTLs) used in breeding programs^150–152^ and, the recent discovery of regulatory genes which influence fruit sugar accumulation^153–158^, are promising targets for improving fruit taste (Table 1). Gene editing for increased sugar may mitigate the loss of tissue sugar content or capacity that occurs due to postharvest handling^69^ and therefore maintain consumer satisfaction, reducing postharvest waste.

#### Starch

The starch-rich organs of cassava, yam (*Dioscorea* spp.), and potato are important staples, but unlike cereal grains, they are highly perishable^159^. There is interest in changing the digestibility of starch to create varieties with different nutritional attributes^160^. For example, low-digestible, i.e., “fiber-like” starch would be healthier upon consumption, and could conceivably resist breakdown in storage, reducing postharvest loss. Transgenic alteration of the starch branching enzymes in potato showed that increasing “resistant starch” could be recapitulated in a horticultural crop^161–168^.

Starch also accumulates in the immature fruit of apples, bananas, tomatoes, and kiwifruit^169^, and its breakdown to sugars at maturity makes valuable contributions to ripe fruit sugar content^151,170^. Increasing starch content by manipulating regulatory proteins and transporters (Table 1), has been identified as a viable strategy for increasing the postharvest quality of ripe fruit^171,172^.

#### Cold-induced sweetening

Potato tubers are stored at low temperatures (4–8 °C) to extend shelf-life and meet industry demand for round-the-year fresh products. However, sucrose and reducing sugars (glucose and fructose) accumulate during cold storage from starch breakdown, a process referred to as cold-induced sweetening (CIS)^173,174^. CIS affects the quality of fried potato products: reducing sugars react with amino acids during high temperature cooking to form carcinogenic acrylamide through the Maillard reaction^175,176^. Several metabolic pathways, including starch biosynthesis and degradation, are involved in CIS^177–180^. Reducing vacuolar invertase activity decreased reducing sugars and alleviated CIS in transgenic tubers^181–185^. These genes are therefore ideal targets for manipulation using a gene-editing approach. Reducing CIS would lessen the severity of tuber postharvest starch loss and would also reduce the postharvest waste that results when blackened chips and fries are discarded.

### Flavor profiles

There seems to be a consensus among consumers that store-bought fruit and vegetables often lack good flavor; one consequence of this assessment is PW. The flavor is determined by the intricate combination of sugars, acids, and volatiles^186^. Improving flavor is made even more challenging because “good flavor” is subjective and varies across and among different consumer populations^187–189^. Postharvest handling and retail systems are major contributors disrupting many of the pathways required for full fruit flavor, especially volatile production^190^. Painstaking efforts have been made to link the abundance of specific chemicals, especially aroma volatiles, to human likeability using sensory panels^191,192^. However, because of the complexity of fruit flavor profiles, targeting multiple genes that affect sweetness, acidity, and aroma is likely necessary to truly improve consumer appeal^193^, a challenge that gene editing may meet more readily than traditional breeding^194^. For example, in tomato, fructose, citric acid, and six aroma volatiles were associated with a high hedonistic value^193^. Novel alleles of genes that contribute to enhanced fruit flavor^194,195^ were related to sugar content, citrate, and volatiles, all of which may be manipulated by gene editing to develop the fruit of optimal flavor^196^.

### Reduced browning

Many horticultural crops exhibit undesirable browning that is a turn-off for consumers who discard these edible but “downgraded” produce^197^. Browning is common in fresh-cut or bruised produce—lettuce, spinach, apples, and potatoes—or is due to physiological disorders such as heat and chilling injury, or exposure to inappropriate oxygen and carbon dioxide levels^197^.

There are two types of browning, enzymatic and non-enzymatic. Non-enzymatic browning describes the Maillard reaction, discussed in the “Cold-induced sweetening” section. Enzymatic browning involves the action of three core enzymes: polyphenol oxidase (PPO), peroxidase (POD), and phenylalanine ammonia lyase (PAL)^197^. Scientists have successfully shown that knocking out PPO genes reduced browning upon wounding. This is a relatively easy target and a proven and effective strategy for reducing postharvest waste^198^. Non-browning, commercially available produce i.e., Innate potato® and Arctic Apple® (Table 1)^185,199,200^ are in retail outlets. Non-browning mushrooms have also been produced by CRISPR–Cas9 gene editing^201^. Manipulation of this trait is expected to make significant inroads in reducing consumer disposal of “browned” but edible produce, especially for those that are “fresh-cut.”

## Complex postharvest traits

There are many postharvest phenotypes that influence the quality and shelf-life of crops that are poorly understood at the molecular level. These traits may be influenced by the activity of multiple genes and their alleles, and how their expression is altered by the environment (see “Identifying genes that influence postharvest traits” section). Our understanding of the disorders that result from these combined elements, along with those discussed in “Composition” section, is hampered by the influence of preharvest factors, which are often not taken into account, and study-to-study variability in experimental design and reporting inconsistencies^69,202^. Postharvest disorders that affect a wide variety of species are discussed below.

### Microbial food safety

*Salmonella* and *E. coli* contamination of fresh-cut fruit, vegetables, and especially leafy greens can occur at various points in production, causing illness or even death if consumed^71,203^. *E. coli* OH157:H7, alone has sickened 72,855 people and led to 173 deaths in the US from 1980–2016 ^204^. These outbreaks have increased in frequency due to (1) intensive farming, (2) the growing complexity of the postharvest supply web, and the (3) popularity of fresh-cut salads, which offer more entry sites for pathogen infestation^204–206^. The problem is that a localized outbreak of a commodity often temporarily suppresses sales and demand, leading suppliers to dump unaffected product, creating waste.

The development of breeding strategies to reduce bacterial attachment, persistence, and proliferation, have the potential to reduce food contamination^203,207^. Identifying genomic regions in *Salmonella* controlling stomatal opening in lettuce leaves^208^, and screening germplasm for tolerance to microbial pathogens are important steps^209^. This type of fundamental knowledge could open new avenues for developing genetic strategies for improved food safety^210^ including gene-editing strategies.

### Postharvest chilling injury

Low temperatures typically extend shelf-life, but in some produce, when rewarmed after chilling, the normal maturation program is disrupted, leading to poor quality^190^. This physiological dysfunction called Postharvest chilling injury (PCI) is manifested in a wide array of symptoms across species, the most severe of which include tissue and seed browning or blackening, pitting, fungal infestation and decay^20,211^, which contribute to postharvest loss. Mild PCI symptoms include a lack of flavor, and undesirable texture and taste^20,211^, which leads to postharvest waste.

We estimate that ~56% of the top 50 global commodities are susceptible to PCI. Further, the symptoms are often hard to specifically ascribe to chilling injury: PCI-accelerated decay is often diagnosed as postharvest disease or premature senescence^212^, and poor flavor induced by PCI is often blamed on variety-type or early-harvest. PCI is insidious because it is difficult to detect, and it is therefore not properly documented^28^. It also adds constraints to postharvest management strategies for sensitive crops, as preventing PCI requires faster shipping at higher temperatures or shorter storage times.

A new gene discovery holds promise for reducing the occurrence of PCI. The *SlGRAS4* gene, when overexpressed in transgenic tomato by RNAi, promoted postharvest chilling tolerance in fruit with no change in yield^213^. *SlGRAS4* overexpression may also be achieved by editing repressor elements in its promoter in the future.

### Preharvest factors

**Fig. 5:**
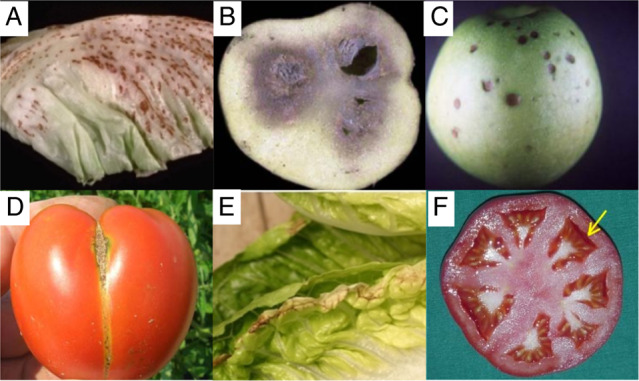
Postharvest disorders in fruit and vegetables. **A** Russet spotting in lettuce, **B** blackheart in potato tuber, **C** bitterpit in apple, **D** zippering in tomato, **E** tipburn in lettuce, **F** puffiness in tomato fruit. Pictures reproduced with kind permission from UC Davis Postharvest Technology Center (**A**, **C**), Marita Cantwell (**B**), Gerald Brust (**D**), Richard Smith (**E**), and Elizabeth Maynard (**F**)

The metabolic and physiological state of a commodity before harvest is a key factor determining its postharvest quality. Soil elemental composition, especially nitrogen and calcium, crop exposure to extreme heat, drought, or even wind, and irregularities in irrigation regime can affect a broad array of quality parameters that may make produce unsuitable for sale (Fig. 3)^214,215^. Visible blemishes such as lettuce tip burn (Fig. 5) are often linked to temperature effects^216^. Still, overwhelmingly, these physiological traits are some of the most difficult to dissect due to the unpredictable nature of the severity and frequency of their occurrence. Identifying genes that could be modified for improvement may be more problematic than for other traits.

### Postharvest storage disorders

Postharvest treatments are used to prolong produce shelf-life (Fig. 2), but incorrect exposure or treatment can disrupt metabolism, leading to physiological disorders occurring in the harvested product^217,218^. These injuries may be initiated at the cellular level due to the overproduction of reactive oxygen species, membrane damage, and energy imbalances caused by interferences in ATP production^219^.

Over time, secondary reactions occur and result in visible changes, e.g., water soaking, tissue browning, blackening, microbial growth and decay, off-flavors, and odors^214,217,219^. Common postharvest disorders include russet spotting in lettuce and blackheart in potato (Fig. 5). The genes underlying these phenotypes are very poorly understood, and studies are complicated because there are multiple interacting factors that result in the trait. The use of -omics technology and the mitigative effects of some hormonal or hormetic physical treatments have led to a better understanding of the signal transduction pathways affected^219^, but more work needs to be done to determine candidates for gene-editing solutions because these traits lead to PLW.

## Roadblocks impeding the broader adoption of gene editing for reducing PLW

Commercial and public implementation of gene editing has been occasional and non-systemic. The reasons for this are manifold and interconnected; however, most stem from two primary issues.

**Fig. 6:**
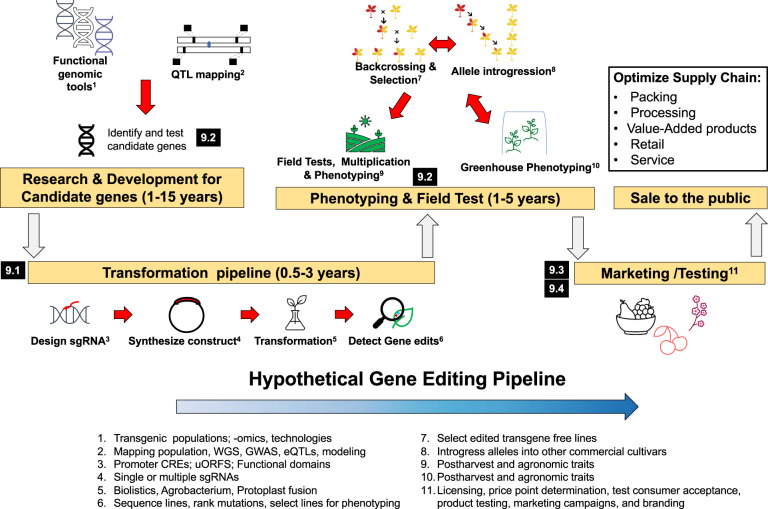
A flowchart with time estimates for developing gene-edited plants. Numbers indicate sections referred to in the text. The more unknowns and undeveloped steps in a potential pipeline for a gene-edited crop, the less appealing beginning that process may be to invested parties, especially given the uncertain and changing regulation

First, many horticultural crops have been traditionally understudied because their lifecycle, genomic structure, or inability to regenerate via tissue culture, are not amenable to methods used in functional genomics. This leads directly to the second reason: many of the genes that contribute to the problems of PLW have not yet been identified. These two identified stumbling blocks will require new and significant financial investments to minimize their effect and accelerate the use of gene editing for reducing PLW (Fig. 6).

### Plant transformation and regeneration

Although transgenic approaches for modifying plants have been advanced for three decades, transformation and regeneration (Fig. 6) are bottlenecks in using gene-editing tools to address crop improvements^220,221^. The efficiency of *Agrobacterium*-mediated approach varies by *Agrobacterium* strain, and the plant species and tissue to be transformed^183^. Almost 95% of woody fruit and nut crops are still recalcitrant to transgenic approaches because of poor transformation efficiencies using *Agrobacterium*^222^. Transformation may be achieved using biolistic and electroporation approaches, which are non-tissue specific^223^ but lead to multiple insertions which can create additional non-intended genetic changes.

Regeneration through tissue culture is even more challenging than the integration of foreign genes, and the process is time-consuming (Fig. 6). Regeneration takes 6–9 months for papaya^224^, 5–8 months for kiwifruit^225^, and 4–5 months for potato^220,226^. Novel delivery approaches to bypass labor-intensive plant regeneration procedures are being developed. “Spray-on” gene editing involves coating nanosized carbon dots with plasmids containing gRNA and Cas9 cDNA, which are delivered directly to the cell^227^. Inducing meristem formation in tissues transformed with the CRISPR gene construct would produce edited plantlets without a callus explant step, thus saving time^228^. Seeds produced from such plants could be propagated^228^. Recent innovations promise to open up the number of crops that can be efficiently modified by gene editing^229,230^. Specifically, transforming calli with a *GRF4-GIF* growth factor chimera has been used to accelerate regeneration efficiency more than 5-fold among some of the most recalcitrant species^229^, and may be a major advance in crop improvement.

### Identifying genes that influence postharvest traits

Many postharvest traits are composite, with phenotypes that are the result of multiple environmental factors as well as genetics (Eq. 1). As mentioned, common disorders such as PCI, blossom end rot, and superficial scald, are challenging to study at the molecular level. These traits are likely to be multigenic and multiallelic, with gene expression controlled in networks involving epistatic interactions and epigenetic mechanisms, that are influenced by environmental factors^231–237^.

**Eq. 1:**
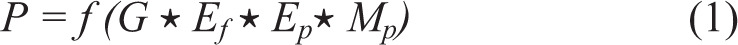
Factors contributing to postharvest phenotype. Where *P* = postharvest phenotype; *f* = function; *G* = genotypic factors; *E*_f_ = field preharvest environment; *E*_p_ = postharvest environment; *M*_p_ = postharvest management. Factors are interactive and presumed to be non-additively, nonlinearly compounding: we use the star operator to suggest the dynamism of a cross-correlation function; though this concept is not typically applied to biological systems, it is evocative here.

For many years, environmental control, i.e., refrigeration and modified and controlled atmospheres, was the primary way of maintaining shelf-life and quality^15^. Forward genetic approaches such as QTL and Association mapping, mutagenesis, and gene expression analysis, have been used to identify candidate genes that exert a significant degree of control over complex agricultural traits^238,239^. However, introgressing favorable genes into a new cultivar may not always lead to the strong expression of a trait, because of environmental effects^240^.

### Patent landscape

There is much uncertainty about the right to market edited germplasm. In the United States, the Broad Institute holds the patent for CRISPR editing of eukaryotic cells^241^. However, the University of California continues to challenge the 2018 ruling on multiple grounds^242–244^. In most of Europe and the Pacific (China, Australia, Singapore), the University of California holds the rights to CRISPR^241^. As shown in Fig. 1, the perishable fruit, vegetable, and ornamental market is global. Those interested in growing “CRISPR’ed” produce in, e.g., California, and selling it in the US, Canada, China, and Australia, might need to invest millions to acquire permissions from both Broad Institute and the University of California^241^. This may increase the entry cost to commercialize postharvest gene-edited products, and limit the traits targeted to those with the highest profit-margin and simultaneously, push out smaller, “boutique” biotechnology firms^241^.

The public sector has not hesitated to use CRISPR^245–247^. The Broad Institute allows unrestricted use of the Intellectual Property covered in its CRISPR patents for non-profit and academic research uses, but commercial planting of the fruits of that research is open to legal challenge^248,249^. Currently, in the US, commercial growers frequently invest in public breeding programs by providing land for field trials to state-level institutions. Material support from for-profit entities may become legally tenuous if a laboratory or facility is using CRISPR or any other patented technology.

As CRISPR techniques become more refined^246,247^ and additional patents are submitted, these legal and financial contingencies may become more labyrinthine. However, the explosion in CRISPR research in plants, shows that the agricultural and horticultural world is eagerly embracing gene editing. The promised profit improvements currently outweigh the potential legal ramifications of patent infringements.

### Regulatory issues

Another roadblock to the commercialization of gene-edited horticultural crops is their differing classification across the globe. The United States and China, which produce and consume a majority of the world’s fruit and vegetables (Fig. 1), have readily embraced gene editing^240^, and regulation in India is based largely on precedent^250^. Modifications produced by gene editing vs. traditional breeding can be functionally identical, and distinguishing said modifications is near impossible^251–253^. As a result, in June 2020 the United States announced the SECURE rule, stating that from April 2021, novel crops with DNA changes that could be introduced by traditional breeding can be fast-tracked for marketing^254^. The European Union, however, has ruled that gene-edited crops are in the same classification as “traditional” GMOs^252,255,256^. This places additional burdens on companies wishing to market edited produce in the EU and UK^255^. European produce markets have high quality standards for flavor and texture^257,258^, and new gene-edited crops could conceivably meet these criteria. The benefits of PLW reductions from gene editing may not be realized as quickly as in Europe.

## Conclusion

Gene manipulation alone cannot solve the problem of horticultural loss and waste, as the overall issue remains heterogeneous and multi-faceted, requiring transdisciplinary advances, and the integration of biological, engineering, and socio-cultural solutions. Consumer awareness campaigns about saving produce are notoriously difficult to develop and implement, and success is variable because human behavior is often intractable. Realizing engineering solutions requires massive long-term investments in infrastructure, equipment, and energy. It is against this backdrop that we explored the potential of gene editing for improving produce to be hardier in the supply chain as well as meet consumer expectations.

Manipulating biological processes by gene editing is both a promising new avenue for reducing PLW and a major challenge that relies on understanding the baroque regulation of these pathways in order to “tweak” them in a beneficial way (Figs. 2,3, and 4). Because the technique is relatively cheap and easy, with minimal impact on the genome (Fig. 6), the cost barrier is such that for the first time, breeders can feasibly engineer postharvest traits with the expectation that the new germplasm could be commercially viable. This means that in spite of the challenges we have outlined (Figs. 5 and 6), there is reason to believe widespread gene editing for PLW reduction is possible and imminent.

**Fig. 7:**
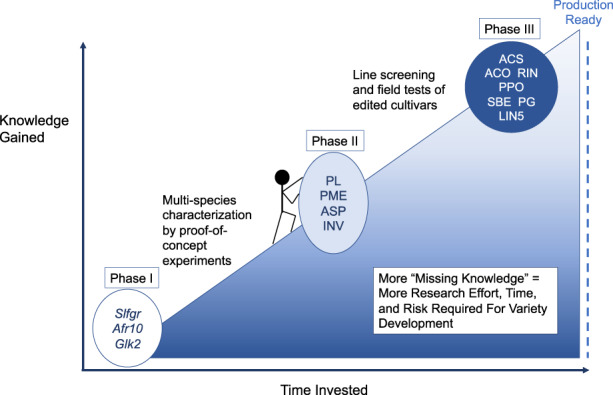
Gene targets for commercialization of novel gene-edited crops. Genes listed in the dark blue oval to the right have been well studied and are likely to have commercially relevant phenotypes, while those in white still require additional testing because the action of these genes have only been demonstrated in a single species. Phase I: *Afr10*—higher fruit sugars; *Glk2*—higher tomato sugars and flavonoids; *Slfgr*—sweeter tomato fruit. Phase II: *INV, ASP* reduced cold-induced sweetening and acrylamide produced during processing in potato; *PME*; *PL*—reduced fruit softening, better storability and consumer acceptance. Phase III: *ACS, ACO, RIN*—reduced rate of ripening; *PG*—reduced fruit softening would cause less bruising during shipment, *PPO*—non-browning, better consumer appeal if bruised postharvest; *LIN5*—higher accumulation of sugars in fruit; *SBE*—higher fiber potato for health

As shown in Fig. 7, several genes have been proven to provide reliable phenotypes for reducing PLW, and there are others that are very promising. Many projects are underway to recapitulate these findings using gene-editing approaches, for extended shelf-life or better quality in major commercial species^85,99,198,201,259,260^. Stacking edited alleles of these genes in crops may also lead to additive or valuable transgressive effects. It is our opinion that gene-edited crops will eventually be in broad use across the globe, because of the clear evidence of their potential to minimize postharvest waste and loss in the context of multiple threats to the stability of the world produce supply chain.
